# First Comprehensive Analysis of Both Mitochondrial Characteristics and Mitogenome-Based Phylogenetics in the Subfamily Eumeninae (Hymenoptera: Vespidae)

**DOI:** 10.3390/insects13060529

**Published:** 2022-06-08

**Authors:** Li Luo, James M. Carpenter, Bin Chen, Tingjing Li

**Affiliations:** 1 Chongqing Key Laboratory of Vector Insects, Chongqing Key Laboratory of Animal Biology, Institute of Entomology and Molecular Biology, College of Life Science, Chongqing Normal University, Chongqing 401331, China; lioly19941112@126.com (L.L.); bin.chen@cqnu.edu.cn (B.C.); 2 Division of Invertebrate Zoology, American Museum of Natural History, Central Park West at 79th Street, New York, NY 10024, USA; carpente@amnh.org

**Keywords:** Eumeninae, gene rearrangement, mitogenomes, phylogenetic, Vespidae

## Abstract

**Simple Summary:**

The subfamily Eumeninae comprises more than 3900 described species and eumenine mitochondrial analyses are almost absent. In order to provide further evidence toward understanding the relationships within the subfamily, the characteristics of 54 eumenine mitogenomes were comparatively analyzed, among which 52 mitogenomes are newly annotated. Meanwhile, using both Maximum-likelihood (ML) and Bayesian inference (BI), comprehensive phylogenetic relationship in the subfamily were investigated based on two mitochondrial datasets.

**Abstract:**

The subfamily Eumeninae plays a significant role in the biological control of agricultural pests. However, the characteristics of eumenine mitogenomes that are important molecular markers for phylogenetics are not clearly revealed. Here, 52 eumenine mitogenomes are newly sequenced and annotated, and the phylogenetic relationships of the subfamily are comprehensively analyzed based on 87 vespid mitogenomes. Through the comparative analysis of the 54 eumenine mitogenomes, the gene compositions of about one half of the 54 species match with ancestral insect mitogenome, and remaining others contain two *trnM* which are highly similar, with 51.86% (*Eumenes tripunctatus*) to 90.65% (*Pseumenes nigripectus*) sequence identities, which is unique among the reported mitogenomes of the family Vespidae. Moreover, the translocation *trnL1* upstream of *nad1* is a common rearrangement event in all eumenine mitogenomes. The results of phylogenetic analyses support the paraphyly of the subfamily Eumeninae and the tribe Odynerini, respectively, and the monophyly of the tribe Eumenini, and verify that the tribe Zethini is a valid subfamily Zethinae. In this study, the relationships between some genera such as *Allorhynchium* and *Pararrhynchium* or the taxonomic status of the subgenera such as *Eremodynerus* and *Dirhynchium* are found to be confusing and there should be further inquiry with more samples.

## 1. Introduction

The subfamily Eumeninae containing more than 3900 described species is the biggest of the family Vespidae (Hymenoptera). It is the primary lineage of the Vespidae [1], which plays a significant role in the biological control of agricultural pests because of its cosmopolitan predation of the larvae of Lepidoptera (e.g., Geometridae, Tortricidae) and Coleoptera (e.g., Chrysomelidae and Curculionidae) [2,3]. Most of them are solitary wasps, using mud to partition the cells, while some are primitively social, burrowing in the soil or wood, many species in Zethini construct their nests by exploiting masticated and salivated plant material such as *Zethus*, *Ischnocoelia*, *Elimus*, *Discoelius,* and *Protodiscoelius* using vegetable matter for cell partition, and *Psiliglossa* and *Raphiglossa* using pith [4,5]. Additionally, the high morphological diversity and complexity of Eumeninae leads to some difficulties in its classification, and some other difficulties may be attributed to its troubled taxonomic history [6]. Hence, the early classifications of this subfamily underwent a radical transformation. Based on the morphology of the mouthparts and the general shape of the metasoma, Latreille began the generic classification of Eumeninae and divided the current species of Eumeninae into three genera: *Eumenes*, *Odynerus*, and *Synagris* [7]. Later, de Saussure divided ‘Euméniens’ into Anomaloptéres, Euptéres, and Mischoptéres, after that he separated *Zethus*, *Calligaster,* and *Discoelius* from the rest of his section ‘Euptéres’ of the ‘Eumhiens’ as the group ‘Zethites’ [8,9]. Thereafter, Richards proposed a classification for the “Eumenidae” with three subfamilies: Raphiglossinae, Discoeliinae (=Zethinae), and Eumeninae [10], while Carpenter did not recognize the names Zethinae and Raphiglossinae after investigating the relationships among the subfamilies of the Vespidae with a cladistic treatment [11]. Recently, Hermes et al. corroborated the monophyly of Eumeninae and proposed three tribes of this subfamily, namely Zethini, Eumenini, and Odynerini, which was the most comprehensive classification of the Eumeninae based on morphology [12]. Whether the tribe Zethini should be upgraded to the subfamily Zethinae and the subfamily Eumeninae is monophyletic are worthy of further exploration. Additionally, with the continuous enrichment of molecular data, controversies over the classifications of Eumeninae appear in the disagreement between morphological and molecular data.

In the published research on phylogenetic relationships in the subfamily Eumeninae, some molecular data have been utilized. The first molecular study showed that the solitary Eumeninae was a sister taxon to the Polistinae + Vespinae cluster leveraged on nuclear 28S rDNA and mitochondrial 16S rDNA of 12 species from the family Vespidae including 3 eumenine species [13]. Thereafter, based on the analysis of four nuclear gene fragments (18S rDNA, 28S rDNA, *abdominal-A,* and *RNA polymerase II*) from 27 Vespidae species (containing 11 eumenine species), Hines et al. supported the division of Eumeninae into two separate monophyletic clades: “Zethinae” and “Eumeninae” [5]. Neither of the two studies clarified the generic relationships of Eumeninae because only a few species of Eumeninae were contained. Later, with a total of 49 transcriptomes of vespid wasps (containing 40 eumenine species), Bank et al. suggested the subfamily Eumeninae was paraphyletic and the “Zethini” were divided into two clades: Raphiglossinae and Zethinae [14]. Then, Piekarski et al. also suggested that “Zethini” should be a valid subfamily Zethinae [15]. So far, several nuclear gene fragments, mitochondrial fragments, and transcriptomes have not completely resolved the phylogenetic relationships of Eumeninae due to insufficient and unrepresentative sampling of taxa. Thus, to understand the evolution of the various biologies exhibited by Eumeninae, robust investigations of phylogenetic relationships are still needed.

The mitogenome is a widely accepted molecular marker used in phylogenetic studies due to its maternal inheritance as well as the higher rate of nucleotide substitution compared with nuclear DNA [16,17]. To date, two mitogenomes of Eumeninae have been published, which is insufficient to explore phylogenetic relationships [18]. In China, which spans two faunal regions (Palearctic and Oriental Regions), there is a total of more than 310 known species and subspecies in 58 genera of the subfamily Eumeninae [19,20,21,22,23,24,25,26,27,28,29,30], which constitutes a quarter of the total genera of the world. To clarify the phylogenetic relationships within Eumeninae, especially the placement of Zethini, 52 new mitogenomes of 33 genera of Eumeninae from China are obtained and analyzed by combining 35 published vespid mitogenomes in this study. Given that gene rearrangements are very informative for phylogenetic analysis and are exhibited extensively in some insect orders [17], these eumenine mitogenomes are compared with the gene order of ancestral mitogenome to investigate their distinctive rearrangement models. Additionally, the characters of the eumenine mitogenomes are compared with other vespids, in order to identify any unique character to support the classification of Eumeninae.

## 2. Materials and Methods

### 2.1. Sample Collection and DNA Extraction

The 52 species of the subfamily Eumeninae, which were firstly identified at least to genus level by taxonomic specialists, were collected and stored in 95% ethanol at −20 °C in Chongqing Normal University, Chongqing, China (Table 1). Thereafter, total DNA was isolated from the muscle tissues of the thorax using the DNeasy DNA Extraction kit (Qiagen) and according to its instructions. Finally, we followed the manufacturer’s protocol of the Qubit dsDNA high-sensitivity kit (Invitrogen) to determine the DNA concentration for each sample.

### 2.2. Whole-Genome Sequencing and Assembling

The Illumina TruSeq library, containing an average size of 350 bp, was sequenced using the Illumina Hiseq 2500 platform at BerryGenomics (Beijing, China). Then, high-quality reads (after deletion of low-quality reads) were used in de novo assembly with IDBA-UD by using the NGS QC Toolkit [31,32]. *COX1* and *srRNA* were amplified by standard PCR reactions and were used to identify mitogenome assemblies with at least 98% similarity sequences in BLAST [33,34]. Finally, the accuracy of the assembly was investigated by mapping clean reads onto the obtained mitochondrial scaffold in each library using Geneious 10.1.3 (http://www.geneious.com/. Accessed date: 12 January 2022), which allowed for up to 2% mismatches, a maximum gap size of 3 bp, and a minimum overlap of 100 bp.

### 2.3. Mitogenome Annotation and Sequence Analysis

Annotation of the assembled mitochondrial sequences was identified using Clustal X 1.8 with homologous sequences against the publicly available Eumeninae mitogenomes [35]. Unrecognized tRNA genes were found by use of tRNA scan-SE version 2.0.2 and secondary structure modeling was completed using ARWEN version 1.2 [36,37]. The nucleotide composition, AT content, GC-skew, and the Relative Synonymous Codon Usage (RSCU) were calculated in MEGA X [38]. Effective Number of Codons (ENC) and GC of silent 3rd codon posit (GC3s) were computed in Codon W 1.4 and non-synonymous (*Ka*) and synonymous (*Ks*) substitution ratio (*Ka/Ks*) of PCGs were calculated in DnaSP 5.0 [39,40]. Then, the gene arrangement events were detected in CREx [41].

### 2.4. Phylogenetic Analyses

A total of 87 Vespidae mitogenomes containing 52 newly sequenced eumenine mitogenomes and 35 species of the subfamilies Eumeninae, Stenogastrinae (three species), Polistinae (19 species), and Vespinae (11 species) downloaded from GenBank were selected as ingroups, and four species from Apoidea (*Hylaeus dilatatus*, *Andrena cineraria*, *Megachile sculpturalis*, and *Apis cerana*) were selected as outgroups (Table S1). In total, 13 PCGs and 2 rRNAs were extracted by PhyloSuite v 1.2.2 [42]. The individual alignments of PCGs were performed using the L-INS-i strategy of the MAFFT algorithm executed in the TranslatorX online platform, and rRNA genes were aligned individually using the G-INS-i strategy implemented in MAFFT version 7.205 [43,44]. GBlocks v.0.91b was used to remove all ambiguously aligned sites from 13 PCGs and two rRNAs [45]. After that, MEGA X was used to check and correct all the alignments [38].

Phylogenetic trees were inferred from two sets of data: (1) PCGR: 13 PCGs and 2 rRNAs; (2) PCG: 13 PCGs. Before the construction of trees, PartitionFinder version 2.1.1 [46] was used to simultaneously choose the best partition schemes and substitution models for each matrix with the Akaike Information Criterion (AIC) and greedy search algorithm (Table S2). A Bayesian inference (BI) tree was constructed in MrBayes v.3.2.7, approximately 10,000,000 generations were conducted for the matrix, with the average deviation of split frequencies below 0.01 which suggests that runs reach convergence and were sampled every 1000 generations with a burn-in of 25% [47]. Maximum likelihood (ML) was constructed on the PhyML online web server (http://www.atgc-montpellier.fr/phyml/. Accessed date: 12 January 2022) and the node support values were evaluated via a bootstrap test with 100 replicates [48]. In addition, for (maximum parsimony) MP, the matrix was analyzed through the use of Winclada slaving TNT [49,50]. New technology search algorithms were used with the default settings, except ratchet 200 iterations, with up:down perturbation 8:4; hits to minimum length 25. Bootstrapping was via traditional search, with 100 replicates.

## 3. Results and Discussion

### 3.1. Mitochondrial Genome Organization

We obtained 52 complete or partial mitogenomes, which were deposited in GenBank (Table 1). Most of them include 13 protein-coding genes (PCGs), 2 rRNA genes (rRNAs), a control region, and 22 or 23 tRNA genes (tRNAs), with a size from 14,241 (*Leptochilus* sp.) to 23,251 bp (*Rhynchium brunneum brunneum*) and some of the entire A+T rich regions as well as three tRNA genes (*trnI*, *trnQ* and *trnM*) were unable to be amplified in 23 mitogenomes (Figure S1). The composition of 29 complete mitogenomes are significantly biased toward adenine and thymine, with high A+T content from 78.6% (*Subancistrocerus camicrus*) to 84.7% (*Eumenes pomiformis*) which is similar to other hymenopteran mitogenomes [51] and the AT skews are from −0.09 (*Pararrhynchium striatum*) to 0.19 (*Antepipona* sp.) (Table S3).

**Figure 1:**
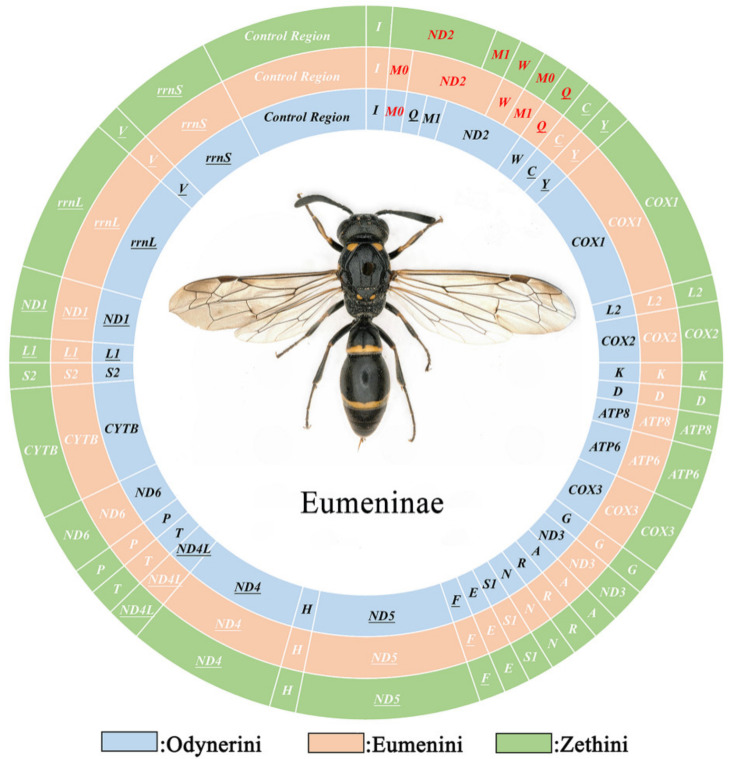
The mitochondrial genomes of Eumeninae. Circles of different colors indicate different tribes of Eumeninae. The red gene means its position is inconsistent with the ancestor insect.

Typically, the mitogenomes of metazoan animals are double-strand circular DNA composed of 37 genes including 13 PCGs, 22 tRNAs, 2 rRNAs, and a control region, and most genes are located on the J-strand (major strand), the remaining being on the N-strand [52]. In this study, some mitogenomes of Eumeninae generally match that of the inferred mitogenomes except for some *trnM* duplications (Figure 1).

**Figure 2:**
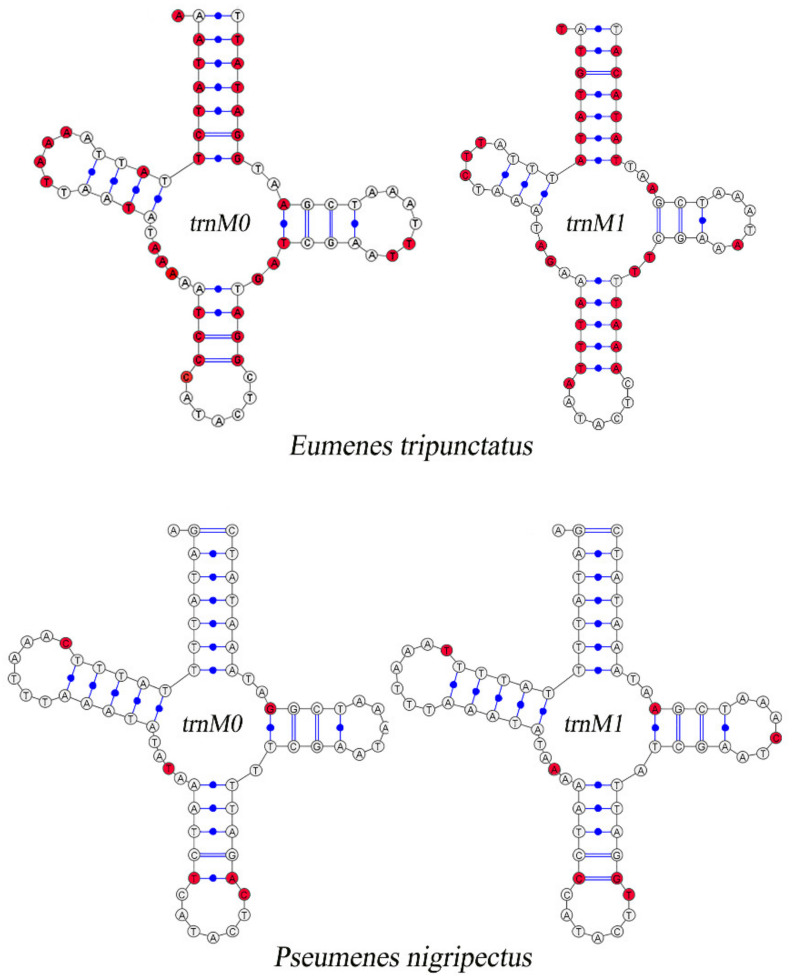
Inferred secondary structures of duplicated *trnM*. The substitutions in *trnM0* and *trnM1* compared with each other are indicated by red color.

Compared with other Vespidae, there are 26 in total in the 52 newly assembled mitogenomes of Eumeninae containing two *trnM* genes which are highly similar, with 51.86% (*Eumenes tripunctatus*) to 90.65% (*Pseumenes nigripectus*) sequence identities (Figure S2). The substitutions between *trnM0* and *trnM1* are identified in the Amino Acid acceptor (AA) arm, TψC (T) arm, Variable (V) loop, Anticodon (AC) arm, and the dihydorouridine (DHU) arm (Figure 2). The positions of *trnM0* and *trnM1* are different: some are connected and others are separated by *trnQ* and *trnW*. The duplication event is unique in the subfamily Eumeninae among the reported mitogenomes of Vespidae; meanwhile, it was reported in the mitogenomes of both *Ibalia leucospoides* (Hymenoptera: Cynipoidea) containing three *trnM* with 92–97% sequence identities and the genus *Pachycephus* (Hymenoptera: Cephidae) [18,53,54]. Moreover, there is another duplication of *trnL2* within the Eumeninae such as three regions of noncoding DNA containing four copies of *trnL2* in *Abispa ephippium* [55]. According to the existing reports, the duplication of tRNA is common in Hymenoptera; for instance, the copies of *trnD*, *trnA*, and *trnE* in the family Cephidae (Hymenoptera) and Trigonalyoidea (Hymenoptera), respectively [18,53]. Therefore, within the family Vespidae, the duplication of *trnM* may be one of the features to indicate whether a species belongs to the subfamily Eumeninae.

### 3.2. Protein-Coding Genes and Codon Usage Patterns

**Figure 3:**
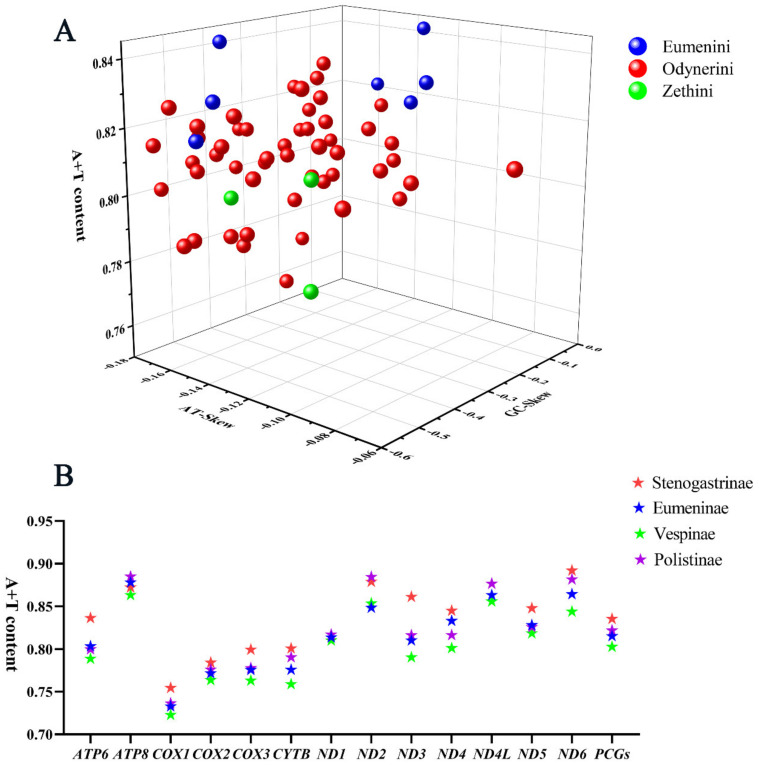
A+T content, AT-Skew, and GC-Skew of PCG in vespid mitogenomes. (**A**) The A+T content, AT-Skew, and GC-Skew of PCG in Eumeninae; (**B**) the A+T content of PCG in four subfamilies of Vespidae.

All the PCGs start with the typical ATA, ATG, or ATT codons and stop with the complete TAA or TAG or truncate TA- or T -- termination codons. The composition of PCGs is significantly biased toward adenine and thymine, with high A+T content from 75.9% to 84.4%, and the AT skews are always negative from −0.16 to −0.095 (Figure 3A). The A+T content of PCGs in other subfamilies of Vespidae was computed, showing that the value of A+T content in Stenogastrinae is higher than in three other subfamilies and in Vespinae it is the minimum (Figure 3B).

**Figure 4:**
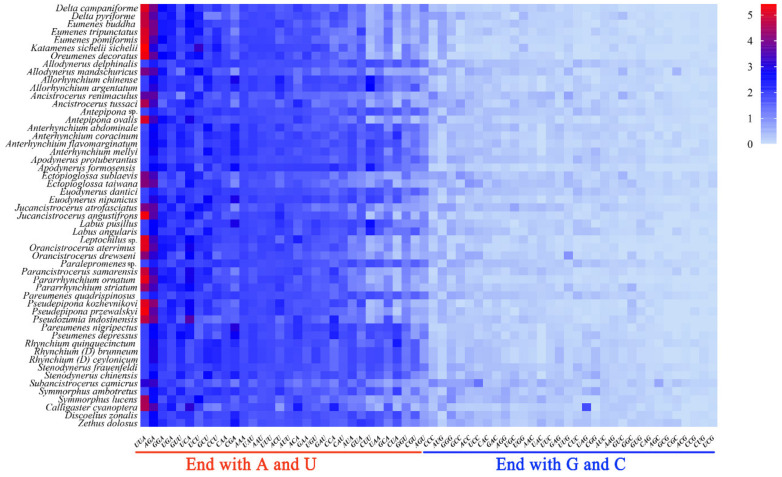
Relative synonymous codon usage (RSCU) of the mitogenomes of Eumeninae.

The Relative Synonymous Codon Usage (RSCU) values of codons such as UUA, GUU which ended with A or U, are all greater than 1.3 and those ending with G or C are all less than 1 (Figure 4). The RSCU value can directly reflect the frequency of codon usage: the RSCU value equivalent to 1 indicates that the codon has no preference, or the RSCU value greater than 1 illustrates that the frequency of the codon is relatively higher [56,57]. As a result, the optimal codons among PCGs of eumenine mitogenomes are codons ending with A or U, and accordingly, the third position of the codon in PCGs is significantly biased toward adenine and thymine with 90.5% A+T content. Additionally, the optimal codons of eumenine mitogenomes are consistent with those of Vespidae which frequently used UUU, UUA, AUU, and AUA, and among them, UUA (Leu2) is the one with the highest RSCU value. In addition, both UAA and AGA are the stop codons of the eumenine mitogenomes, of which UAA with the higher RSCU is the eumenine preferred codon.

**Figure 5:**
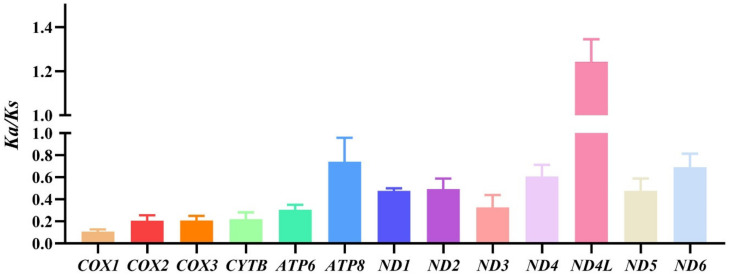
The *Ka/Ks* values of the subfamily Eumeninae are based on each PCG.

The synonymous codon usage bias is influenced by mutation pressure and natural selection, and an effective number of codons (ENC) standard curve can indicate that the determinant of codon preference is mutation pressure or natural selection [58]. Our result shows that all the points lie under the standard curve, which indicates that the codon usage bias is influenced by selection pressure (Figure S3). *Ka/Ks* is the ratio of the number of nonsynonymous substitutions per nonsynonymous site (*K*a) to the number of synonymous substitutions per synonymous site (*K*s), which could indicate something about the selective forces acting on the protein [59]. Thus, we computed the *Ka/Ks* value of PCGs from eumenine mitogenomes, and the result shows that all the *Ka/Ks* of PCGs except *ND4L* are less than 1, which indicates that only *ND4L* is under a positive selection and evolves rapidly, and other PCGs are under a purifying selection. Moreover, the lowest *Ka/Ks* value of *COX1* (0.11) indicates that it is conservative under environmental selection pressure and suitable for molecular barcoding (Figure 5).

### 3.3. Gene Arrangement

**Figure 6:**
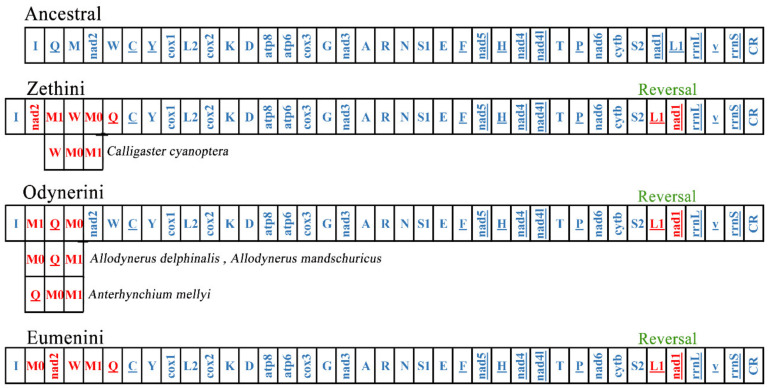
The rearrangement event in three tribes of the subfamily Eumeninae. The red genes represent its’ position changes compared with ancestral mitogenomes.

Mitogenomes are usually stable in composition and gene arrangement is relatively conservative; therefore, recombination rarely occurs in the evolutionary history of insects [52,60]. As more and more mitogenomes of insects are reported, the rates of mitogenome rearrangement in Hymenoptera are accelerated [53,61]. The subfamily Eumeninae is the primary lineage of the Vespidae, and its gene rearrangement events are still poorly studied. Some eumenine mitogenomes contain a duplication of *trnM* and the positions of the two *trnM* are different, which means that the different mechanisms occurred in the gene rearrangement of Eumeninae. We investigated more rearrangement events of 54 eumenine mitogenomes and found that all eumenine mitogenomes contain a translocation *trnL1* upstream of *nad1* (Figure 6). Because gene duplications are not allowed in CREx, the rearrangement events in cluster *trnQ*-*trnM*-*ND2*-*trnW* are inferred as three patterns compared with the ancestral mitochondrial gene order (Figure S4): the tandem duplication of *trnM* occurs in all three clusters of Eumeninae, and then the distinct recombination occurs in the three clusters, respectively. In the tribe Zethini, the recombination occurs in *trnQ-trnM0* and *trnM1*-*ND2* after *trnM* duplicated to *trnM0*-*trnM1*, and subsequently, it occurs between *trnM0*-*trnQ* and *ND2*-*trnM1*-*trnW*, and there is another rearrangement type: from the ancestral order *trnQ*-*trnM*-*ND2-trnW* to *ND2*-*trnW-trnM0*-*trnM1* in *Calligaster cyanoptera*. In the tribe Odynerini, the recombination between *trnM1* and *trnQ-trnM0* occurs in most species, and the recombination *trnQ-trnM0* after *trnM* duplicated to *trnM0*-*trnM1* occurs in *Allodynerus delphinalis* and *Allodynerus mandschuricus*. In the same way, the recombination occurs in *trnQ-trnM1* after *trnM* duplicated to *trnM0*-*trnM1*, and then the recombination occurs between *trnM1 trnQ* and *trnM0*-*ND2-trnW* in most species of the tribe Eumenini. As mentioned above, the three tribes of the subfamily Eumeninae possess their distinctive rearrangement pattern. Our results provided additional evidence that the majority of mitogenome rearrangements occur in tRNAs in hymenopteran insects and also showed that the gene block *trnI*-*trnQ*-*trnM*-*ND2* may be the hot spot of rearrangement in Hymenoptera because the rearrangement events of this block are found in many hymenopteran lineages, such as the rearrangement events of the gene block *CR*-*trnI*-*trnQ*-*trnM*-*ND2*-*trnW*-*trnC*-*trnY* in all the Icheumonoid lineages and rearrangement of *CR*-*trnI*-*trnQ*-*trnM* in the mitochondrial genome of *Allantus luctifer* [62,63].

### 3.4. Phylogenetic Relationship of Vespidae

**Figure 7:**
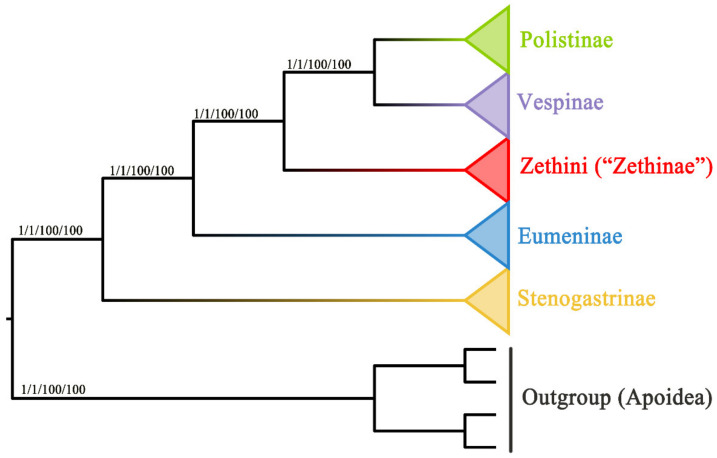
Phylogenetic trees of the Vespidae inferred from PCG and PCGR by ML and BI. Each node shows the Bayesian posterior probabilities (PP)/maximum likelihood bootstrap support (BS) values.

The results of substitution saturation show that Iss > Iss.c and *p* = 0.0000 within both PCG and PCGR (Table S4), which indicates that the sequences are not saturated and can be used for phylogenetic analysis. In this study, phylogenetic analyses of two concatenated nucleotides (PCG and PCGR) were conducted, both representing four subfamilies (Stenogastrinae, Eumeninae, Polistinae, and Vespinae) of Vespidae and the outgroup (*Hylaeus dilatatus*, *Andrena cineraria*, *Megachile sculpturalis*, and *Apis cerana*). The two concatenated nucleotides were subjected to Bayesian inference (BI) and maximum likelihood (ML) analyses, resulting in four trees where the positions of the four subfamilies are congruent (Figure 7). In these trees, the phylogenetic relationships of the Vespidae are as follows: Stenogastrinae + (“Eumeninae” + (Zethini + (Polistinae + Vespinae))). According to our results, the subfamilies Stenogastrinae, Polistinae, and Vespinae are undoubtedly monophyletic, but nevertheless, the subfamily Eumeninae excluding Zethini is monophyletic. Additionally, Stenogastrinae is a sister lineage to other subfamilies of Vespidae with high bootstrap support values (BS) and Bayesian posterior probabilities values (PP) (BS = 100, PP = 1), which is consistent with some recent phylogenomic studies [64,65]. The study reveals that the tribe Zethini of the subfamily Eumeninae is an independent branch and more closely related to Polistinae and Vespinae, which is similar to previous studies [5,13,15]. As the tree shows in Figure 7, the position of the Zethini (“Zethinae”) is between solitary Eumeninae and eusocial Vespinae + Polistinae. The genus *Calligaster* and the subgenus *Zethoides* of genus *Zethus* in Zethini (“Zethinae”) have been cited as exemplifying the critical evolutionary stages of subsocial and communal behavior which connects solitary and eusocial wasps because it is reported that some species of both *Zethus* and *Calligaster* construct their nests with plant material rather than the typical eumenine nest construction with mud [66,67].

### 3.5. Phylogenetic Relationship within Eumeninae

**Figure 8:**
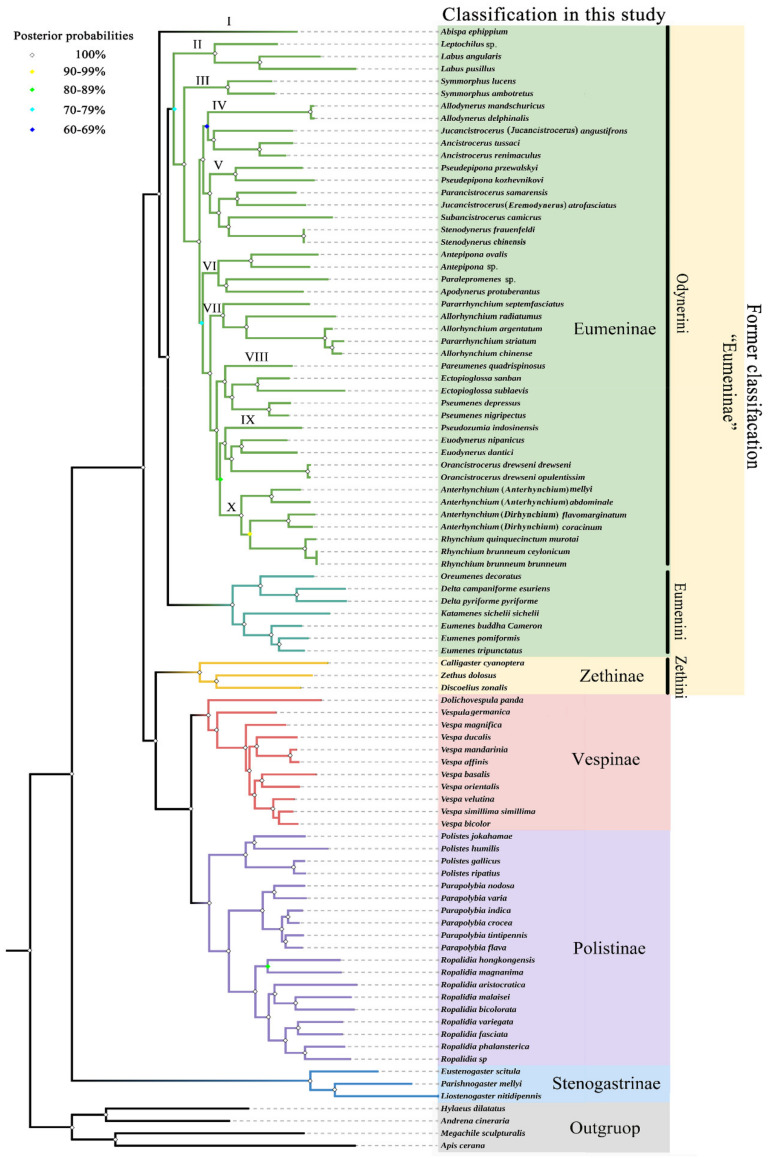
Phylogenetic tree of Vespidae inferred from PCG and PCGR by BI. Each node shows the Bayesian posterior probability (PP) values.

The ingroup relationships of the family Vespidae are congruent in the obtained trees with the same methods, respectively, and the notable difference between the obtained tree topologies with ML and BI methods is that *Pseudozumia indosinensis* belongs to clade VIII in ML trees with low bootstrap support value (BS = 39, 38), while in BI trees it belongs to clade IX with high Bayesian posterior probability (PP = 1,1) (Figure S5). That the BS of a branch is lower than 50 means that the relationship has not been supported. In order to verify the accuracy of the obtained trees, a MP analysis was also performed, and the results were (of course) similar to the BI trees (Figure S6). Therefore, with the high Bayesian posterior probabilities, it is more likely that *Pseudozumia indosinensis* belongs to clade IX in BI trees. Of course, it may be because the differing placement in the ML trees is an artifact of bootstrap values below 50. The placement of the genus *Pseudozumia* in BI trees is also consistent with the result of Piekarski et al. In their research, a maximum-likelihood tree of Vespidae inferred from 235 selected loci obtained also shows the genus *Pseudozumia* as a sister group to *Orancistrocerus* which belongs to clade IX in this study [15]. Furthermore, the illustration in Figure 8 is identical to the results from analyzing the data of PCG and PCGR with the BI method. Both ML and BI reveal that the tribe Eumenini is monophyletic and the tribe Odynerini is paraphyletic containing 10 clades. The results also consistently indicate that the clade II to clade X is the sister group to the tribe Eumenini.

Within the tribe Eumenini, the sister relationship of (*Oreumenes* + *Delta*) + (*Katamenes* + *Eumenes*) is strongly supported by all datasets in this study (BS = 100; PP = 1). Hermes et al. found that the genera *Oreumenes*, *Delta*, *Katamenes,* and *Eumenes* all belong to their clade 3 of Eumenini, and the genus *Eumenes* was recovered as a sister to the remaining taxa of Eumenini [6]. The differences in phylogenetic relationships of the four genera between our study and Hermes et al. might be attributed to our limited generic sample, which is not enough to clarify the comprehensive relationships of all genera of the tribe Eumenini. Therefore, to clearly understand the phylogenetic relationships within the tribe Eumenini, more data are needed.

The tribe Odynerini is the biggest one within the subfamily Eumeninae [6]. Here, we investigated 24 genera of Odynerini to illustrate their phylogenetic relationships. The results show that Odynerini comprising 10 major clades (I-X) is paraphyletic, and clade I (the genus *Abispa*) is a sister group to all remaining Eumeninae. Bank et al. reported that the genus *Alastor* (clade A) is inferred as a sister lineage to all remaining Eumeninae based on transcriptomes of 49 vespid wasps [14]. Our study does not contain any species in the genus *Alastor*, and Bank *et al*.’s study did not contain any species in the genus *Abispa*, whereas that of Piekarskis et al. containing both *Abispa* and *Alastor*, is consistent with the standpoint of Bank et al. [14,15]. In clade II, the genus *Leptochilus* is inferred as a sister lineage to the genus *Labus*. In succession, the genus *Symmorphus* is an independent clade III and sister group to clades IV-X. In clades IV-X, there is a sister-group relationship between clades IV-V and VI-X. Within clades IV-V, clade IV is a sister group to clade V, while *Jucancistrocerus* (*Jucancistrocerus*) *angustifrons* and *Jucancistrocerus* (*Eremodynerus*) *atrofasciatus* are located at clades IV and V, respectively, which may support subgenera *Eremodynerus* being a valid genus [68]. Of course, more morphological evidence of more species should be investigated to confirm our results in further research. Within clades VI-X, clade VI is a sister group to clades VII-X and is composed of *Antepipona +* (*Paralepromenes* + *Apodynerus*). Then, clade VII is a sister group to clades VIII-X, composed of *Pararrhynchium* and *Allorhynchium*, while *Pararrhynchium striatum* is located within the genus *Allorhynchium*. The misidentification of *Pararrhynchium striatum* is eliminated by the examination of specimens, so *Pararrhynchium striatum* should be transferred to the genus *Allorhynchium*, or these two genera are synonymized. Likewise, there is a sister relationship between clade VIII and clades IX-X, and the phylogenetic relationships of clade VIII are as follows: (*Pareumenes* + (*Ectopioglossa* + *Pseumenes*)). Finally, within these two clades IX and X, they are sisters to each other, and the phylogenetic relationships of clade IX are as follows: (*Pseudozumia* + (*Euodynerus + Orancistrocerus*)), and of clade X they are (*Anterhynchium* (*Anterhynchium*) + (*Anterhynchium* (*Dirhynchium*) + *Rhynchium*)). As the results show, with high Bayesian posterior probabilities (PP = 1), the subgenus *Dirhynchium* of *Anterhynchium* is more closely related to the genus *Rhynchium* than the nominate subgenus *Anterhynchium*, which means the subgenus *Dirhynchium* should be upgraded to a valid genus. Again, further investigation is needed.

## 4. Conclusions

To sum up, the mitogenomes of Eumeninae are commonly found to contain two *trnM*, which differs remarkably from the gene orders of other Vespidae. This study based on mitogenomes further supports previously proposed relationships among Vespidae [5,14,68], especially the placement of the tribe Zethini and some genera of the subfamily Eumeninae, indicating that the tribe Zethini should be raised to Zethinae and that the tribe Eumenini is monophyletic and Odynerini is paraphyletic. Meanwhile, some issues have not been clearly resolved in this study. First, stable generic morphological characters are needed to support these two subgenera *Eremodynerus* and *Dirhynchium* as valid genera. Additionally, although *Pararrhynchium striatum* is proposed to be moved to *Allorhynchium*, it is possible that the relationship between these two genera is confused, which requires more species sampling and morphological characteristics to elucidate. Second, considering that only one limited mitogenome in some genera of Eumeninae, such as *Abispa*, *Apodynerus*, *Leptochilus*, *Parancistrocerus*, *Paralepromenes*, *Pareumenes, Pseudozumia,* and *Subancistrocerus*, is presented in our analyses, the taxonomic status of these genera may be unstable and uncertain. In the end, the relationships of these taxa in this study still need to be verified by morphological and biological information. Therefore, to further advance the research on the systematic relationships of the subfamily Eumeninae, more taxon sampling and information about the morphological characteristics, molecular data, and biological behaviors are needed.

## Figures and Tables

**Table 1:** GenBank accession numbers for mitogenomes of Eumeninae newly sequenced and annotated in this study.

Species	bp	Gene Number	Accession No.
*Allodynerus delphinalis*	16,932	38	ON024142
*Allodynerus mandschuricus*	17,449	38	ON012816
*Allorhynchium chinense*	16,909	38	MK051021
*Allorhynchium argentatum*	17,972	38	MK051022
*Allorhynchium radiatumus*	17,349	38	ON055163
*Ancistrocerus renimaculus*	15,614	36	ON045342
*Ancistrocerus tussaci*	15,679	35	ON012815
*Antepipona* sp.	19,040	38	ON012817
*Antepipona ovalis*	17,514	36	ON012818
*Anterhynchium abdominale*	16,488	36	MK051029
*Anterhynchium coracinum*	16512	36	MK051028
*Anterhynchium flavomarginatum*	15,196	35	MK051026
*Anterhynchium mellyi*	18,692	38	ON012812
*Apodynerus protuberantus*	17,943	36	ON045341
*Calligaster cyanoptera*	16,316	38	ON012814
*Delta pyriforme pyriforme*	14,883	35	ON076029
*Delta campaniforme esuriens*	16,126	36	ON055486
*Discoelius zonalis*	15,435	38	ON076025
*Eumenes buddha*	16,048	37	ON076024
*Eumenes tripunctatus*	15,702	36	ON045343
*Eumenes pomiformis*	16,520	38	ON076031
*Ectopioglossa sublaevis*	16,203	36	ON045340
*Ectopioglossa sanban*	16,454	37	ON012813
*Euodynerus dantici*	17,493	37	ON076022
*Euodynerus nipanicus*	22,088	38	ON076021
*Jucancistrocerus atrofasciatus*	18,848	38	ON045348
*Jucancistrocerus angustifrons*	19,867	38	ON012819
*Katamenes sichelii sichelii*	14,807	37	ON076027
*Labus pusillus*	17,409	36	ON076026
*Labus angularis*	15,227	35	ON076030
*Leptochilus* sp.	14,241	36	ON045339
*Orancistrocerus drewseni drewseni*	17,636	38	ON045338
*Oreumenes decorates*	15,563	37	ON076028
*Paralepromenes* sp.	16,805	37	ON045337
*Parancistrocerus samarensis*	17,773	38	ON076023
*Pararrhynchium striatum*	20,403	38	ON045347
*Pararrhynchium septemfasciatus*	18,003	36	ON055487
*Pareumenes quadrispinosus acutus*	17,426	38	ON076020
*Pseudepipona kozhevnikovi*	15,650	35	ON076019
*Pseudepipona przewalskyi*	20,281	37	ON024141
*Pseudozumia indosinensis*	16,376	35	ON045335
*Pseumenes nigripectus*	17,773	38	ON045336
*Pseumenes depressus*	16,677	38	ON045346
*Rhynchium quinquecinctum murotai*	16,317	36	MK051030
*Rhynchium brunneum brunneum*	23,251	38	MK051031
*Rhynchium brunneum ceylonicum*	23,122	38	MK051032
*Stenodynerus frauenfeldi*	17,252	36	ON045334
*Stenodynerus chinensis*	17,194	38	ON045345
*Subancistrocerus camicrus*	18,035	38	ON045344
*Symmorphus ambotretus*	17,280	38	ON076018
*Symmorphus lucens*	17,865	38	ON076017
*Zethus dolosus*	16,306	38	ON076016

## Data Availability

Mitochondrial genome sequences are accessible on GenBank and accession numbers are contained within Table 1.

## Supplementary Materials

The following supporting information can be downloaded at: https://www.mdpi.com/article/10.3390/insects13060529/s1, Supplementary Figure S1. The gene order of eumenine mitogenomes used in this study; Supplementary Figure S2. Inferred secondary structures of duplicated *trnM*. The substitutions in *trnM0* and *trnM1* compared with each other are indicated by red color; Supplementary Figure S3. ENC-GC_3S_ plot of total PCGs in 54 eumenine mitogenomes, the black curve shows the relationship between ENC values and GC_3S_ under random codon usage assumption; Supplementary Figure S4. The hypothesized pathway of the translocated inversion derived by recombination and duplication in three tribes of the subfamily Eumeninae. The red genes represent their positions; Supplementary Figure S5. Phylogenetic trees of Vespidae were inferred from PCG and PCGR by ML. Each node shows the bootstrap support values; Supplementary Figure S6. Phylogenetic trees of Vespidae. (A): Phylogenetic tree of Vespidae inferred from PCG by MP. (B): Phylogenetic tree of Vespidae inferred from PCGR by MP. Each nod shows the bootstrap support values; Supplementary Table S1. Mitochondrial genomes used for phylogenetic analysis in this study; Supplementary Table S2. The best partitioning scheme selected by PartitionFinder for different data matrices; Supplementary Table S3. Base composition, total length (bp), and AT-skew of complete Eumeninae mitogenomes; Supplementary Table S4. Substitution saturation test results.
