# Supplementary material for: First Comprehensive Analysis of Both Mitochondrial Characteristics and Mitogenome-Based Phylogenetics in the Subfamily Eumeninae (Hymenoptera: Vespidae)
Source: Insects. 2022 Jun 8;13(6):529. doi: 10.3390/insects13060529 (PMC9225260; doi:10.3390/insects13060529)
Supplement: Supplementary file 1 [file insects-13-00529-s001.zip › insects-1755276-supplementary.pdf]

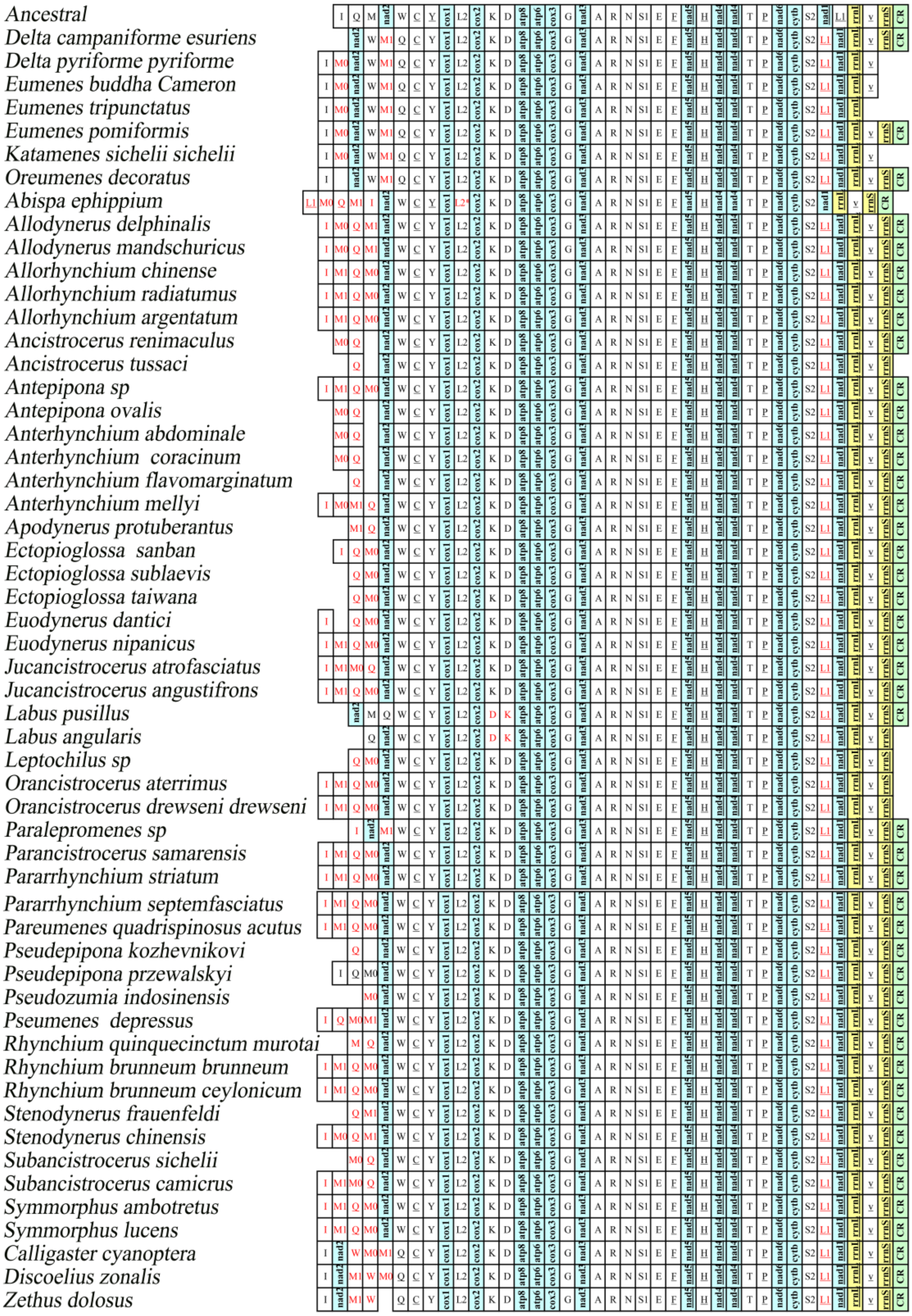

Figure S1. The gene order of eumenine mitogenomes used in this study.

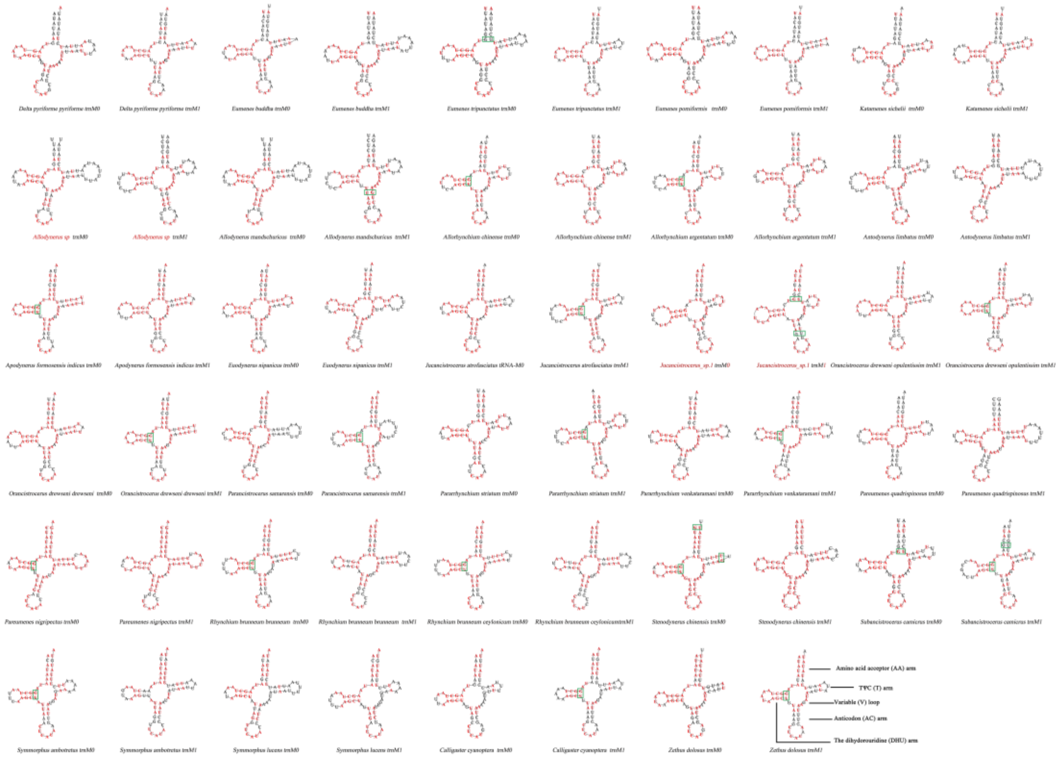

Figure S2. Inferred secondary structures of duplicated *trnM*. The substitutions in *trnM0* and *trnM1* compared with each other are indicated by red color.

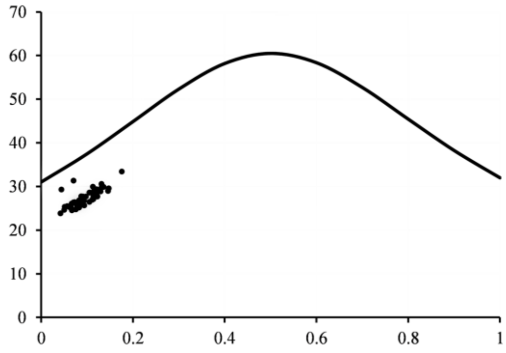

Figure S3. ENC-GC$_{3S}$ plot of total PCGs in 54 eumenine mitogenomes, the black curve shows the relationship between ENC values and GC$_{3S}$ under random codon usage assumption.

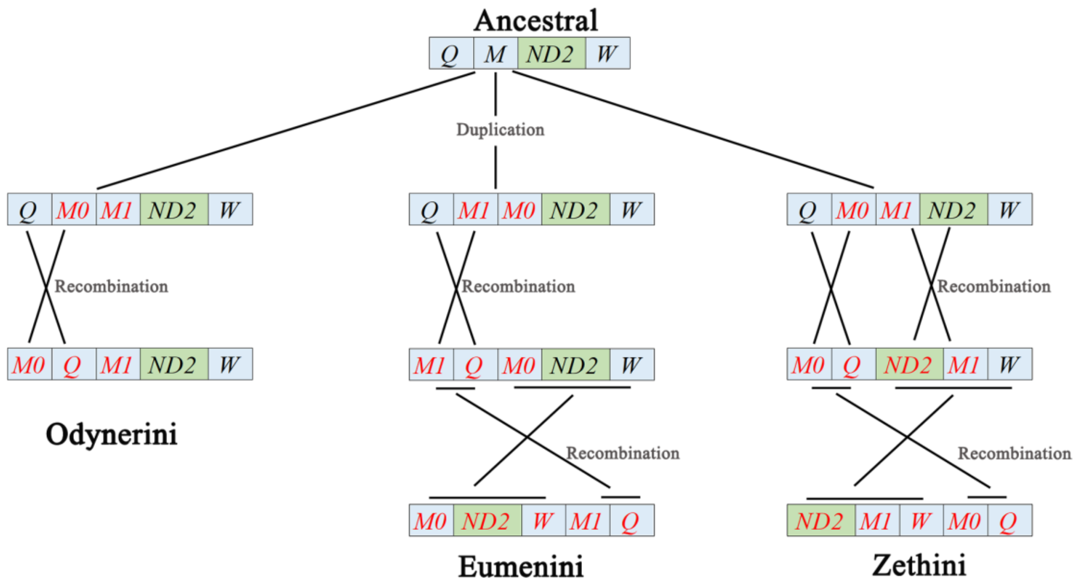

Figure S4. The hypothesized pathway of the translocated inversion derived by recombination and duplication in three tribes of the subfamily Eumeninae. The red genes represent their positions changed.

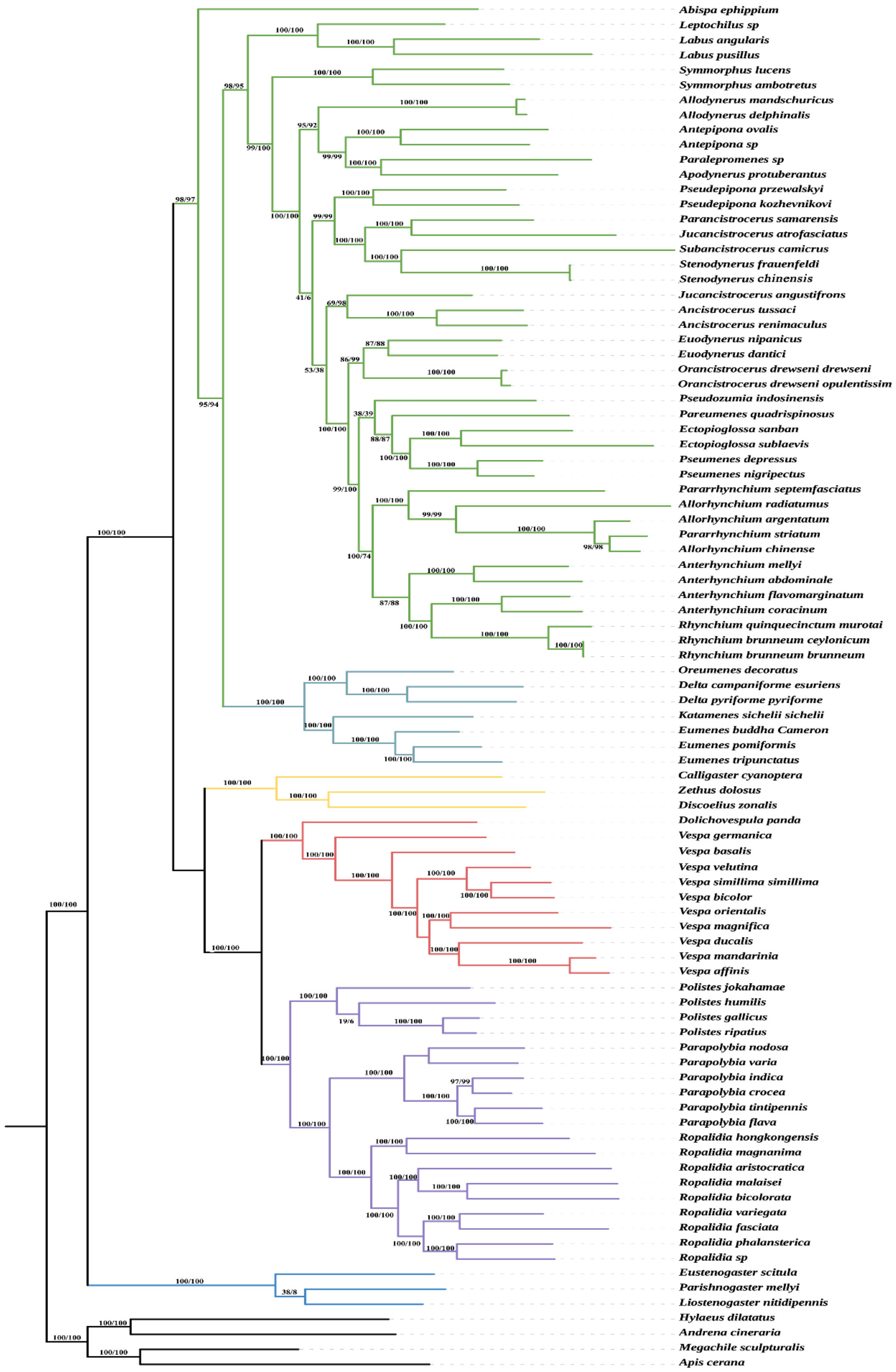

Figure S5. Phylogenetic trees of the Vespidae inferred from PCG and PCGR by ML. Each nod shows the bootstrap support (BS) values.

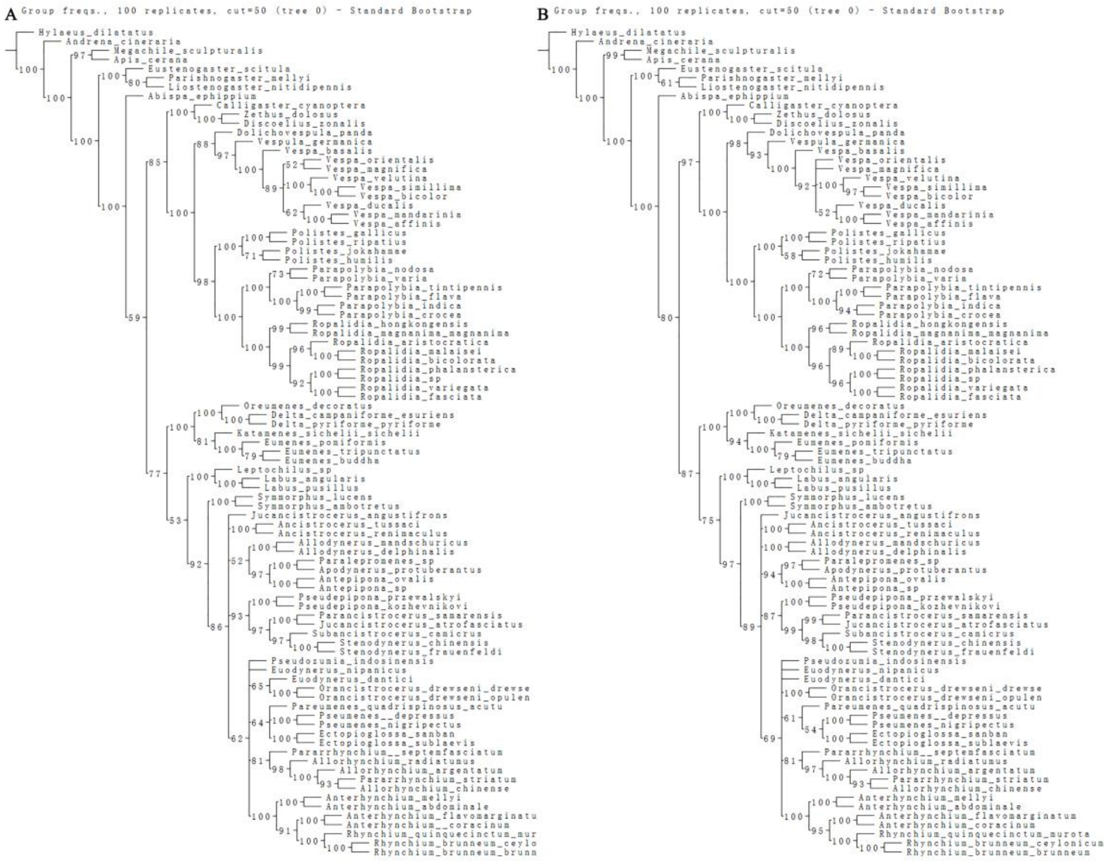

Figure S6. Phylogenetic trees of the Vespidae. A): Phylogenetic tree of the Vespidae inferred from PCG by MP. B): Phylogenetic tree of the Vespidae inferred from PCGR by MP. Each nod shows the bootstrap support values.

Table S1. Mitochondrial genomes used for phylogenetic analysis in this study.

Family	Subfamily	Species	Accession No.	Group
**Vespidae**	Stenogastrinae	*Eustenogaster scitula*	KY856830	Ingroup
		Liostenogaste rnitidipennis	MH910629	
		Parishnogaster mellyi	MH910630	
	Vespinae	*Vespula germanica*	KR703583	
		Vespa mandarinia	NC_027172	
		Vespa bicolor	KJ735511	
		Vespa velutina	NC_035146	
		Vespa orientalis	KY563657	
		Vespa magnifica	MT137097	
		Vespa ducalis	KX950825	
		Vespa basalis	MK440075	
		Vespa affinis	NC_039134	
		Vespa simillima simillima	NC_046020	

		Dolichovespula panda	NC_036067	
	Polistinae	*Ropalidia* sp	MK034142	
		Ropalidia phalansterica	MK034143	
		Ropalidia bicolorata	MK034144	
		Ropalidia fasciata	MK034145	
		Ropalidia magnanima	MK034146	
		Ropalidia hongkongensis	MK034147	
		Ropalidia variegata	MK034148	
		Ropalidia malaisei	MK034149	
		Ropalidia aristocratica	MK034150	
		Parapolybia varia	MH065751	
		Parapolybia tintipennis	MH065754	
		Parapolybia flava	MH065755	
		Parapolybia nodosa	MH065756	
		Parapolybia indica	MH065757	
		Parapolybia crocea	KY679828	
		Polists humilis	EU024653	
		Polistes jokahamae	KR052468	
		Polistes riparius	LC519884	
		Polistes gallicus	ON017795	
	Eumeninae	*Abispa ephippium*	EU302588	
		Allodynerus delphinalis	ON024142	
		Allodynerus mandschuricus	ON012816	
		Allorhynchium chinense	MK051021	
		Allorhynchium argentatum	MK051022	
		Allorhynchium radiatumus	ON055163	
		Ancistrocerus renimaculus	ON045342	
		Ancistrocerus tussaci	ON012815	
		Antepipona sp	ON012817	
		Antepipona ovalis	ON012818	
		Anterhynchium abdominale	MK051029	
		Anterhynchium coracinum	MK051028	
		Anterhynchium flavomarginatum	MK051026	
		Anterhynchium mellyi	ON012812	
		Apodynerus protuberantus	ON045341	
		Calligaster cyanoptera	ON012814	
		Delta pyriforme pyriforme	ON076029	
		Delta campaniforme esuriens	ON055486	
		Discoelius zonalis	ON076025	
		Eumenes buddha Cameron	ON076024	
		Eumenes tripunctatus	ON045343	
		Eumenes pomiformis	ON076031	
		Ectopioglossa sublaevis	ON045340	

		Ectopioglossa sanban	ON012813	
		Euodynerus dantici	ON076022	
		Euodynerus nipanicus	ON076021	
		Jucancistrocerus atrofasciatus	ON045348	
		Jucancistrocerus angustifrons	ON012819	
		Katamenes sichelii sichelii	ON076027	
		Labus pusillus	ON076026	
		Labus angularis	ON076030	
		Leptochilus sp	ON045339	
		Orancistrocerus aterrimus aterrimus	KY941926	
		Orancistrocerus drewseni drewseni	ON045338	
		Oreumenes decorates	ON076028	
		Paralepromenes sp	ON045337	
		Parancistrocerus samarensis	ON076023	
		Pararrhynchium striatum	ON045347	
		Pararrhynchium septemfasciatus	ON055487	
		Pareumenes quadrispinosus acutus	ON076020	
		Pseudepipona kozhevnikovi	ON076019	
		Pseudepipona przewalskyi	ON024141	
		Pseudozumia indosinensis	ON045335	
		Pseumenes nigripectus	ON045336	
		Pseumenes depressus	ON045346	
		Rhynchium quinquecinctum murotai	MK051030	
		Rhynchium brunneum brunneum	MK051031	
		Rhynchium brunneum ceylonicum	MK051032	
		Stenodynerus frauenfeldi	ON045334	
		Stenodynerus chinensis	ON045345	
		Subancistrocerus camicrus	ON045344	
		Symmorphus ambotretus	ON076018	
		Symmorphus lucens	ON076017	
		Zethus dolosus	ON076016	
**Apoidea**	Megachilidae	*Megachile sculpturalis*	NC028017	Outgroup
	Andrenidae	*Andrena cineraria*	KT164628	
	Colletidae	*Hylaeus dilatatus*	NC026468	
	Apidae	*Apis cerana*	NC014295	

Table S2. The best partitioning scheme selected by PartitionFinder for different data matrices.

Data matrices	Optimal Partition	Model	Initial Partition
PCG-codon partition	Partition 1	GTR+I+G	*a6p1, c1p1, cbp1, c3p1, a8p1, c2p1, n1p1, n5p1, n4p1, n4lp1*
	Partition 2	GTR+I+G	*c1p3, n3p3, c2p3, c3p3, cbp3, a6p3, a8p3, n1p3, n4p3, n5p3*
	Partition3	GTR+I+G	*a8p2, n4p2, n1p2, n5p2, a6p2, n3p2, c1p2, c2p2, c3p2, cbp2*
	Partition 4	GTR+G	*n2p1, n6p2, n2p2, n3p1, n6p1, n4lp2, n2p3, n6p3, n4lp3*
PCGR-codon partition	Partition 1	GTR+I+G	*c3p2, cbp2, a6p2, a8p2, n4p2, n1p2, c1p2, n3p2, c2p2, n5p2*
	Partition 2	GTR+I+G	*a6p1, c3p1, a8p1, c2p1, c1p1, cbp1, n1p1, n5p1, n4p1*
	Partition 3	GTR+I+G	*c3p3, cbp3, a6p3, a8p3, n4p3, n1p3, c1p3, n3p3, c2p3, n5p3*
	Partition 4	GTR+ G	*n3p1, n6p1, n4lp1, n2p1, n6p2, n2p2, n4lp2, n6p3, n4lp3, n2p3*
	Partition 5	GTR+G	*rrnl, rrnS*

a6/8: atp6/8, c1/2/3: cox1/2/3, n1/2/3/4/4l/5/6: nd1/2/3/4/4l/5/6, cb: cytb

Table S3. Base composition, total length (bp) and AT-skew of complete Eumeninae mitogenomes.

Species	bp	A	C	G	T	GC%	AT%	AT-skew
Allodynerus delphinalis	16932	40.3	11.7	5.9	42.1	17.6	82.4	-0.022
Allodynerus mandschuricus	17449	40.3	11.5	5.9	42.2	17.4	82.5	-0.023
Allorhynchium chinense	16909	39	11.1	6.1	43.8	17.2	82.8	-0.058
Allorhynchium argentatum	17972	38.8	11.5	6.3	43.4	17.8	82.2	-0.056
Antepipona sp	19040	41.6	11.7	6.7	40	18.4	81.6	0.020
Anterhynchium mellyi	18692	39.9	11.8	7	41.4	18.8	81.3	-0.018
Calligaster cyanoptera	16316	40.9	11.9	6.4	40.8	18.3	81.7	0.001
Discoelius zonalis	15435	41.5	14.2	6.6	37.7	20.8	79.2	0.048
Eumenes pomiformis	16520	41.8	9.8	5.5	42.9	15.3	84.7	-0.013
Ectopioglossa sanban	16454	41.1	12.3	6.8	39.7	19.1	80.8	0.017
Euodynerus dantici	17493	40	10.2	6.7	43.1	16.9	83.1	-0.037
Euodynerus nipanicus	22088	39.1	9.8	7.7	43.4	17.5	82.5	-0.052
Jucancistrocerus atrofasciatus	18848	37.7	15	7.5	40.2	22.5	77.9	-0.032
Jucancistrocerus angustifrons	19867	39.5	9.3	6.6	44.6	15.9	84.1	-0.061
Oreumenes decorates	15563	42.4	9.5	5.9	42.2	15.4	84.6	0.002
Parancistrocerus samarensis	17773	39.5	11.3	6.3	42.9	17.6	82.4	-0.041
Pararrhynchium striatum	20403	37.1	10.5	8.4	44	18.9	81.1	-0.085
Para.septemfasciatus	18003	39.6	11.8	8.3	40.3	20.1	79.9	-0.008
Pareumenes quadrispinosus	17426	40.9	11.6	8.2	39.2	19.8	80.1	0.021
Pseudepipona przewalskyi	20281	38.1	8.8	8.4	44.7	17.2	82.8	-0.080
Pseumenes nigripectus	17773	39.6	11.4	5.9	43.1	17.3	82.7	-0.042
Pseumenes depressus	16677	43.2	11.2	6.1	39.6	17.3	82.8	0.043
Rhynchium brunneum	23251	36.7	9.3	7.7	46.3	17	83	-0.116
Rh. brunneum ceylonicum	23122	36.8	9.4	7.6	46.2	17	83	-0.113
Stenodynerus chinensis	17194	40.1	12.8	6.3	40.9	19.1	81	-0.010
Subancistrocerus camicrus	18035	39	14.7	6.7	39.6	21.4	78.6	-0.008
Symmorphus ambotretus	17280	40.2	14.3	6.5	39	20.8	79.2	0.015
Symmorphus lucens	17865	40	13.7	6.3	40.1	20	80.1	-0.001
Zethus dolosus	16306	41.1	13.3	6.5	39.1	19.8	80.2	0.025

Table S4. Substitution saturation test results.

Data partition	Iss	Iss.cSym†	Psym‡	Iss.cAsym§	Pasym¶
PCG	0.387	0.7532	0.0000	0.5684	0.0000
PCG+RNA	0.342	0.7489	0.0000	0.5640	0.0000

†Index of substitution saturation assuming a symmetrical true tree.

‡Probability of significant difference between Iss and Iss.cSym (two-tailed test).

§Index of substitution saturation assuming an asymmetrical true tree.

¶Probability of significant difference between Iss and Iss.cAsym (two-tailed test).
